# Multi-Omics Dataset of Heat Stress-Induced Memory in Arabidopsis

**DOI:** 10.1038/s41597-026-07813-z

**Published:** 2026-10-19

**Authors:** Venkatesh P. Thirumalaikumar, Liang Yu, Devender Arora, Umrah Mubeen, Andrew Wiszniewski, Dirk Walther, Patrick Giavalisco, Saleh Alseekh, Andrew D. L. Nelson, Aleksandra Skirycz, Salma Balazadeh

**Affiliations:** 1 Boyce Thompson Institute, Cornell University, Ithaca, NY 14850 USA; 2 Proteomics facility, Purdue University, West Lafayette, IN 47907 USA; 3 Max Planck Institute of Molecular Plant Physiology, Am Mühlenberg 1, 14476 Potsdam-Golm, Germany; 4 Department of Botany and Plant Pathology and Center for Plant Biology, Purdue University, West Lafayette, IN 47907 USA; 5 Metabolomics Core Facility, Max Planck Institute for Biology of Ageing, Cologne, Germany; 6 Center of Plant Systems Biology and Biotechnology, Crop Quantitative Genetics, 4023 Plovdiv, Bulgaria; 7 Department of Biochemistry, Michigan State University, East Lansing, USA; 8 Institute of Biology, Leiden University, Sylviusweg 72, 2333 BE Leiden, The Netherlands; 9 Present Address: University of Göttingen, Albrecht-von-Haller-Institute for Plant Sciences, Julia-Lermontowa-Weg 3, D-37077 Göttingen, Germany

**Keywords:** Plant stress responses, Abiotic

## Abstract

In their natural environment, plants experience temperature fluctuations, including intensified heat waves driven by climate change, which pose significant threats to their productivity. To adapt, plants have evolved diverse mechanisms to withstand heat stress (HS), minimizing potential damage and ensuring survival. One such adaptation is acquired thermotolerance (AT), in which prior exposure to HS primes plants to withstand subsequent severe HS. AT can persist for several days and involves a recovery period during which plants establish heat stress memory (HSM), reorganize cellular processes, and strengthen stress resilience. The molecular mechanisms underlying HSM remain an active area of investigation. This dataset provides high-throughput comparative multi-omics data, including the transcriptome, metabolome, and proteome of *Arabidopsis thaliana* seedlings at distinct intervals during the HSM phase. As the first detailed multi-omics dataset of the HSM phase in Arabidopsis seedlings, it serves as a valuable resource for future research aimed at identifying regulators of heat stress resilience and advancing the development of heat-tolerant crops.

## Background & Summary

As sessile organisms, plants have developed numerous mechanisms to perceive and respond to fluctuating environmental conditions. In their natural habitats, stressors such as drought, temperature extremes, and salinity frequently arise and recur throughout their life cycle^1–4^. To cope with such stressful conditions, plants develop acquired tolerance through priming, where prior stress exposure enhances their ability to mount a more efficient response to subsequent stress events. Priming triggers a new cellular homeostasis state that can persist during the recovery period, even after the priming stimulus is removed. This state enhances the plant’s preparedness for future stress events, a phenomenon known as memory^1^. Evidence shows that stress-priming followed by the establishment of memory is beneficial for stress survival in plants, particularly in the short term^5–11^.

High temperatures adversely affect plants’ growth and productivity^3^. Plant cells redirect their metabolism and resources to mitigate or prevent heat stress (HS)- induced damage. Prolonged exposure to HS disrupts proteome integrity through protein misfolding and/or denaturation, leading to cell toxicity and death^12–14^. To cope with HS, plants activate their heat stress response (HSR) pathways, such as (i) increasing their antioxidant activities, (ii) activating and/or repressing HS-related transcription factors (TFs) in conjunction with the accumulation of heat shock proteins (HSPs)^3^, adjusting their chromatin architecture through activation or repression of the epigenetic regulators^1,13,14^, among other strategies.

HSPs are grouped into different families based on their molecular masses: HSP70, HSP90, HSP100/ClpB (Hsp101), and small HSP (sHSP) families. Small HSPs with monomeric molecular mass ranging from 12 to 42 kDa belong to an evolutionarily conserved family characterized by a common α-crystalline domain located at the C-terminal part^15^. Some of these sHSPs are targeted to different cellular compartments, including chloroplasts, mitochondria, cytoplasm, and the endoplasmic reticulum (ER), with several studies confirming their role in protecting organelle-specific proteins during or after HS^16–18^.

Cellular and molecular connections related to plant growth and stress defense are well described using biological networks. Often, these biological networks are constructed based on one or two omics strategies, which may not provide a holistic view of the responses in context^19^. It is increasingly recognized that a better understanding of biological processes can be achieved through multi-omics approaches. Multi-omics studies offer a more comprehensive platform for deciphering the mechanisms and pathways involved in various biological models^19,20^.

While numerous studies have described the HS responses during or shortly after HS in Arabidopsis, there has been a lack of integrated multi-omics research focusing on events during the HS recovery period and the establishment of memory. Here, we present the first detailed multi-omics dataset of the heat stress memory (HSM) phase in Arabidopsis seedlings, providing insights into the multifaceted nature of this complex process.

**Fig. 1:**
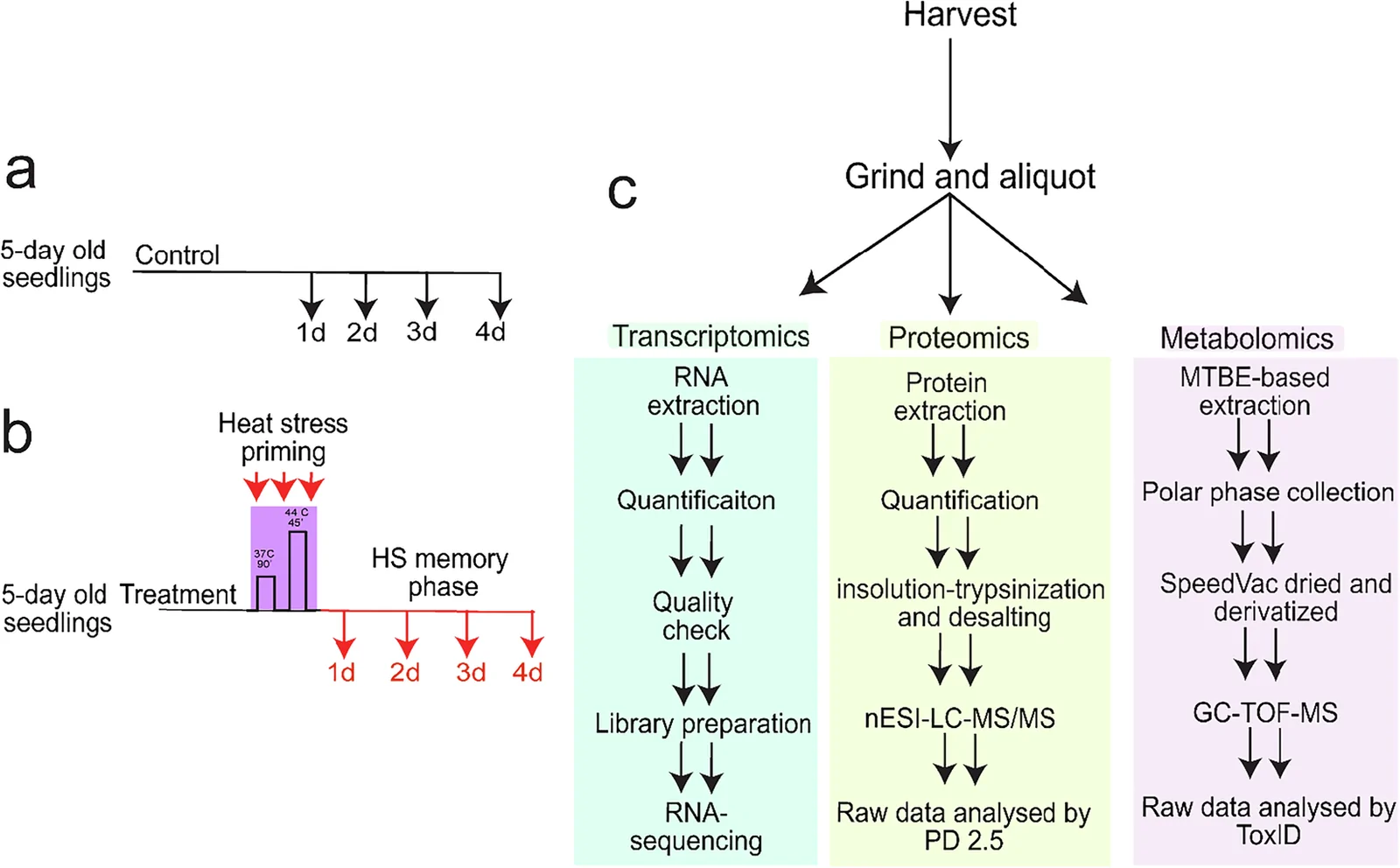
Graphical illustration of the experimental setup and sample collection. (**a**) Seedlings not subjected to HS priming treatment but experienced the same light regime changes were harvested as control seedlings at the indicated time points. (**b**) Five-day-old *Arabidopsis* *thaliana* seedlings were subjected to a two-step HS priming regime of 37 °C for 90 min, followed by recovery at room temperature for 90 min, and then 44 °C for 45 min. After the priming stimuli, the seedlings were returned to regular growth conditions. The seedlings were harvested at different time points as plants entered the HS memory/HSM phase, as indicated. (**c**) Schematic representation of the experimental pipeline. Seedlings were ground using liquid nitrogen conditions and aliquoted for RNA extraction, proteomic, and metabolomic analysis. For proteomics and metabolomics, MTBE-methanol method^22,36^ was used. Polar fractions were analyzed using GC-MS, while protein fractions were analyzed using LC-MS/MS.

Figure 1 shows the experimental conditions and pipeline employed for various omics approaches (transcriptome, proteome, and metabolite profiling). We use the term HSM phase to describe the period during which a plant recovers from HS priming while simultaneously consolidating and retaining the memory of that HS. We employed multi-omics approaches such as untargeted proteomics, transcriptomics, and metabolomics. These datasets will identify the important molecular signatures during the HSM phase in Arabidopsis by comparing the proteomes of primed and control plants.

This paper presents a dataset on gene expression, protein profiles, and metabolic changes during the formation of HSM in plants, providing a resource for advancing research to enhance crop resilience to rising temperatures due to climate change.

## Methods

### Plant materials and growth conditions

*Arabidopsis thaliana* ecotype Col-0 seeds were surface sterilized and sown on Petri dishes containing an equal amount of Murashige and Skoog (MS) 0.8% agar medium supplemented with 1% sucrose (w/v). Seeds were stratified at 4 °C in the dark for 2 days. Seedlings were grown in growth chambers experiencing a 16 h light/8 h dark cycle at 22 °C.

### Heat priming treatment of Arabidopsis seedlings and sample collection

All priming heat stress treatments reported in the study were performed in Petri dishes as described^8–10,21^. For the priming heat stress, five-day-old seedlings were subjected to a heat regime of 1.5 h at 37 °C (incubator), 1.5 h recovery at 22 °C, and 45 min at 44 °C (hot water bath). After the priming heat stress treatment, seedlings were returned to normal growth conditions for 4 days (termed the heat stress memory (HSM) phase), during which samples were harvested for analyses. Five-day-old seedlings of Arabidopsis (Col-0) were harvested after heat priming treatment, particularly during the HSM phase (as indicated in Fig. 1), and subsequently ground in liquid nitrogen. Controls without priming treatment were also harvested at the same time points. Whole seedlings were harvested at the same time of day to minimize circadian effects and ground in liquid nitrogen for subsequent extractions. The same starting biological material was used for proteomics, metabolomics, and transcriptomics analyses, with transcriptomics performed on aliquots from the same sample batch.

### Total protein extraction and in-solution trypsin digestion for LC-MS/MS analysis

Phase separation and total protein extraction were performed as described by^22^. For protein extraction, the solvent-phase separated protein pellet was dissolved in extraction buffer containing (6 M urea, 2 M thiourea, 15 mM DTT, and protease and phosphatase inhibitors). Protein extracts were clarified by centrifugation (10,000 x g, 5 min), and 50 μg of the resulting supernatant was digested with a Trypsin/Lys-C mixture (Promega) following Bradford quantification. After desalting using C18 StageTips, the peptide mixtures were analyzed by LC-MS/MS on an EASY-nLC 1000 system coupled to a Q-Exactive Plus mass spectrometer (Thermo Fisher Scientific). System stability and mass accuracy were monitored throughout the run using standards from the HeLa and cytochrome c digests.

### Label-free quantitative shotgun mass spectrometry and protein identification

A Q-Exactive (Thermo Fisher Scientific, Dreieich, Germany) mass spectrometer coupled to an EASY-nLC 1000 system (Thermo Fisher Scientific, Dreieich, Germany) was used for mass spectrometric proteomic analysis. Methods adapted from^23^. In brief, 1-2 µL of the sample solution was injected onto a 2 cm, C18, 5 µm, 100 Å reverse phase trapping column (Acclaim PepMap100, Thermo Fisher Scientific, Dreieich, Germany) at a flow rate of 4 µLmin^−1^. Peptide separation was done on a 50 cm, C18, 3 µm, 100 Å reverse phase analytical column (Acclaim PepMap100, Thermo Fisher Scientific, Dreieich, Germany). Peptides were eluted by a non-linear 5–30% (v/v) acetonitrile gradient in 0.1% (v/v) formic acid at a flow rate of 300 nL min^−1^ over 90 min, at 35 °C.

Transfer of eluted peptides into the mass spectrometer was performed using a nano electrospray ionization (NSI) source (Thermo Fisher Scientific, Dreieich, Germany) equipped with stainless steel nano-bore emitters (Thermo Fisher Scientific, Dreieich, Germany). Spray voltage was set to 2.4 kV, capillary temperature to 275 °C, and S-lens RF level to 50%. The MS was run in positive ion mode, and MS/MS spectra (top 10) were recorded. Full MS scans were performed at a resolution of 70,000, whereas 17,500 was used for MS2 scans. Automatic gain control (AGC) targets for MS and MS/MS were set to 1 × 106 and 1 × 105, respectively. Only peptides with 2, 3, or 4 positive charges were considered.

### Proteomic data processing

Raw data were processed using the Proteome Discoverer (version 2.5, Thermo Fisher Scientific) software using the Arabidopsis protein database (Version 10, The Arabidopsis Information Resource, www.arabidopsis.org). An in-house list of common contaminants was added to the search. SEQUEST HT was used to assign the peptides, allowing a maximum of 2 missed tryptic cleavages. Additionally, a minimum peptide length of six amino acids, a precursor mass tolerance of 10 ppm, and a fragment mass tolerance of 0.02 Da were selected. Carbamidomethylation of cysteines and oxidation of methionine were specified as static and dynamic modifications, respectively. False discovery rates (FDRs) of 0.01 and 0.05 validated peptide spectral matches at high and medium confidence, respectively. Label-free quantification (LFQ) based on MS1 precursor ion intensity was performed in Proteome Discoverer with a minimum Quan value threshold set to 0.0001 for unique peptides. The ‘Top 3N’ peptides were used for area calculation. The normalized protein abundances were used among the samples, and values were log2-transformed and imputed using a normal distribution pattern. RStudio and Microsoft Excel were used to plot graphs.

### RNA extraction and sequencing

RNA was extracted from Arabidopsis seedlings during heat stress memory (HSM) phase and appropriate control conditions as described previously^21,24^. Briefly, 100 mg of liquid nitrogen ground samples were used to extract total RNA using TRIzol reagent, followed by purification with the Ambion RNA extraction kit (Invitrogen), following the manufacturer’s instructions. DNase I (1U/µL; Thermo Fisher Scientific) treatment was performed following the manufacturer’s protocol. The integrity of the RNA was verified using an RNA gel, and the concentration was measured using a NanoDrop spectrophotometer.

### Transcriptome sequencing and data processing

RNA samples from four biological replicates of seedlings harvested under control and during the heat stress memory phase were sequenced on the Illumina HiSeq. 4000 platform (100 bp single end reads). Library preparation and sequencing were performed by BGI Genomics, China (http://www.bgi.com/). Sequencing adaptors and low-quality bases were removed using Trimmomatic v0.38^25^, and reads shorter than 25 bp were discarded. Filtered short reads were then aligned to the Arabidopsis reference genome using Hisat2 with default settings, and the gene-level transcription abundance was quantified using featureCounts^26^, parameter: (-t exon, -g gene ID, -s 0) based on the latest gene annotation of Arabidopsis^27^. To ensure accuracy, only uniquely mapped reads were retained to avoid multiple counting of ambiguously aligned reads. Gene expression profiling was performed using the DESeq 2 package in R/Bioconductor^28^. Quantified reads of wild-type samples comprising two treatments, four timepoints (day 1 to day 4), and two to four replicates were initially normalized using the DESeq function. Pairwise comparison of gene expression between the priming and control conditions at each time point (day 1~day 4) was then performed to identify differentially expressed genes (DEGs). DEGs were identified using an adjusted *P*-value cutoff of <0.05 and an absolute log_2_ fold change ≥ 2. Normalized counts were then transformed using variance stabilizing transformation (VST), and the top 10% genes showing the highest variation across the wild-type samples were retained to characterize the expression profile. The RNA-seq raw reads have been deposited in the NCBI BioProject database (www.ncbi.nlm.nih.gov/bioproject) under the accession ID SUB15359361^29^.

### Metabolite extraction

Primary metabolites were extracted from ground seedling material following the protocol described by^22,30^. Briefly, approximately 50 mg of frozen, ground plant tissue was resuspended in 1 mL of pre-cooled (−20 °C) methyl tert-butyl ether followed by phase separation using 700 μL of UPLC-grade water/methanol (3:1 v/v) and centrifugation. After removal of the organic phase, 200 μL of the polar phase was aliquoted to a fresh 1.5 mL Eppendorf tube and dried in a SpeedVac. The dried samples were derivatized, and primary metabolites were analyzed by GC-TOF-MS as described by^31^. Raw GC-MS files were processed using the pipeline described by^32^. Further data analysis was conducted using RStudio.

## Data Records

RNA-seq reads and their corresponding peak files have been submitted to the National Center for Biotechnology Information (NCBI) Sequence Read Archive (SRA) under accession number SRP589115^29^. The proteomic raw data have been submitted to the MASSIVE repository under accession number MSV000098061^33^. The metabolomic raw data from the corresponding samples have been submitted to MASSIVE under accession number MSV000098074^34^.

## Technical Validations

**Fig. 2:**
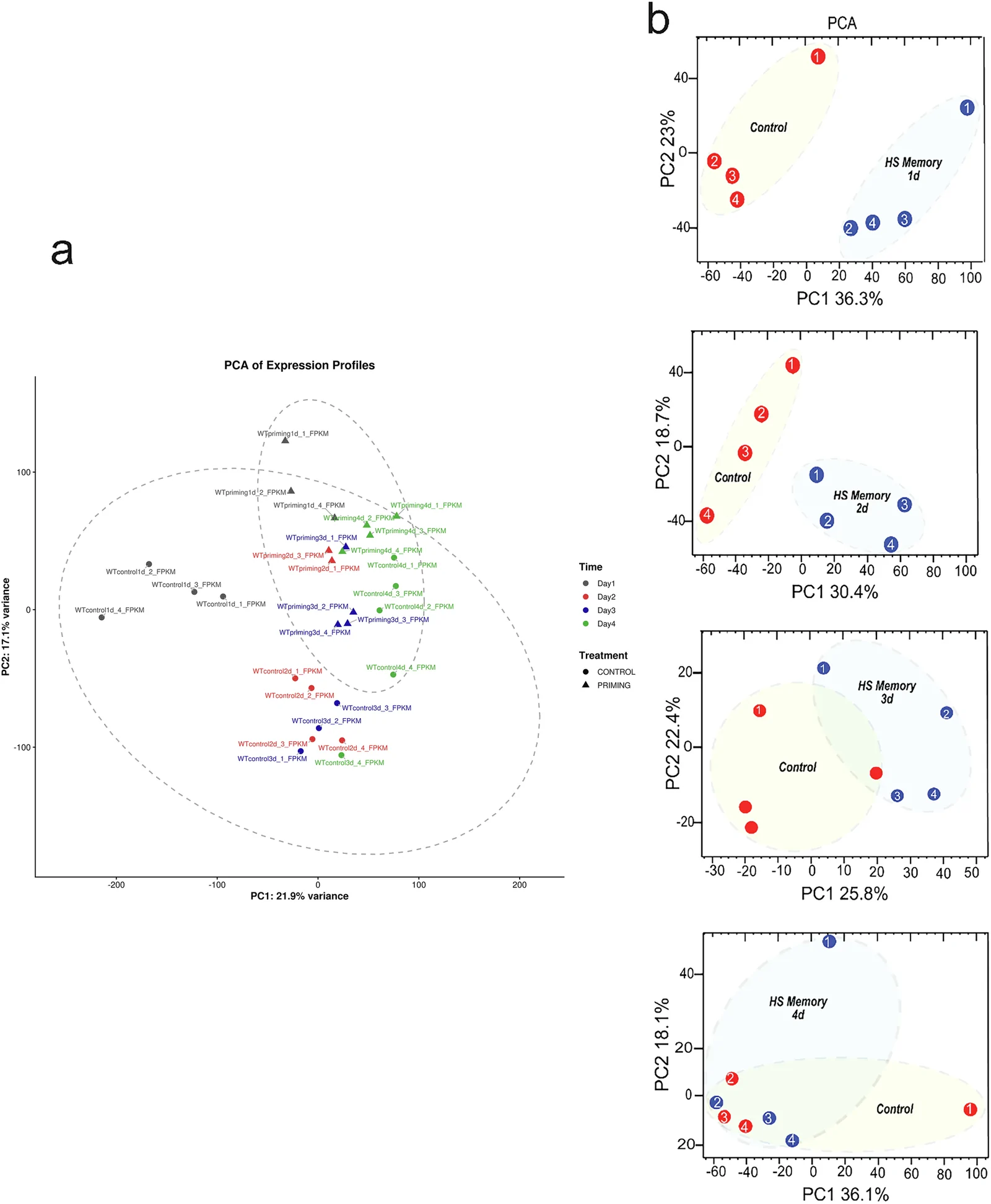
Overview of sample relationships in transcriptomic and proteomic datasets during the HSM phase. (**a**) Principal component analysis (PCA) of transcriptomic profiles across the same set of samples and conditions. Samples are distributed according to overall expression patterns across time points and treatments. (**b**) Principal component analysis (PCA) of proteomic profiles across control and heat stress-primed samples collected at different time points during the HSM phase. Numbers 1–4 indicate individual biological replicates for each condition, and axes indicate the percentage of total variance explained by the principal components.

**Fig. 3:**
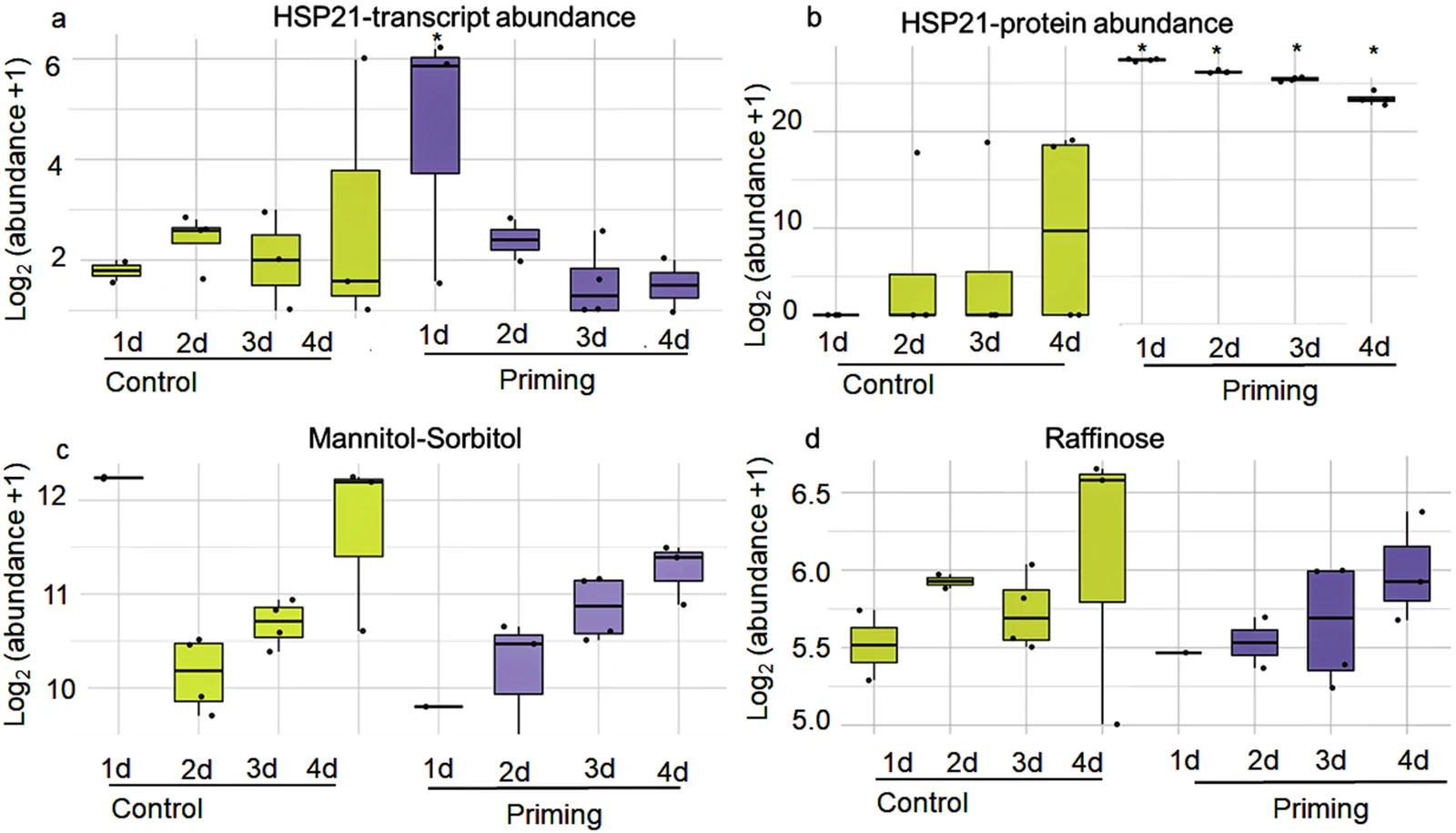
Validation of the transcriptomics, proteomics, and metabolomic dataset for Arabidopsis seedlings during the HSM phase. (**a**) The box plot depicts the transcript abundance of the *HSP21* (*Heat shock protein 21*) at various time points after priming and their appropriate time-specific controls. The mRNA levels of HSP21 are high during the priming phase. (**b**) The box plot depicts the protein abundance of the HSP21 at various time points after priming and their appropriate time-specific controls. The protein levels of HSP21 are high during HSM. Note that the HSP21 protein levels are not detected or detected at very low levels under control conditions; the normal distribution-based imputation was applied to control protein values. (**c,d**) Normalized levels of raffinose family oligosaccharides during the heat stress memory phase in Arabidopsis are presented as box plots. Panel (**c**) shows mannitol/sorbitol, and panel (**d**) shows raffinose. The x-axis represents different treatments (priming or control conditions), while the y-axis displays normalized values on a log scale. Data represents the means of two to four biological replicates ± SD. Asterisks indicate significant difference (Student’s *t*-test; *, *P* ≤ 0.05) from controls. Days (**d**) represent after priming.

Validation analyses were conducted to assess the quality and integrity of the multi-omics datasets under heat stress. In total, ~5000 proteins, ~18000 transcripts, and 250 metabolites were identified in our experiment. Notably, metabolites and proteins were co-isolated from the same biological material using an MTBE-based protocol, while transcriptomic analysis was performed on an aliquot of the same starting plant material. The transcriptome datasets were evaluated for quality and integrity of the datasets using PCA. As shown in Fig. 2a, the high PCA score and clear separation of samples indicate the robustness of the datasets and reflect biological variations across different days of the HSM phase. To validate the transcriptome data, we examined the expression of the heat-stress memory marker gene *HSP21*. Notably, HSP21exhibited low expression under control conditions, whereas its transcript levels were strongly induced one day after the priming stimulus and remained elevated, *albeit* at somewhat reduced levels, at later time points during the HSM (Fig. 3a). This expression pattern is consistent with a sustained transcriptional response characteristic of HSM.

The proteomics data revealed a clear distinction between the control and priming stimulus conditions during the heat stress memory (HSM) phase, as expected (Fig. 2b). To further validate the dataset, we examined the abundance of HSP21 during HSMs. HSP21 was undetectable or present at low levels under control conditions but was strongly upregulated during the HSM phase (Fig. 3b). This is consistent with previous reports showing that HSP21 maintains high abundance during the HSM phase for at least 48 hours^8^. The observed patterns in our dataset support the robustness and biological relevance of the proteomic dataset.

The metabolomics datasets were validated by cross-referencing with previously published reports on heat stress priming in Arabidopsis^35^. It is important to note that to date, no time-course studies have examined the metabolomic response across multiple time points during the HSM phase. A previous study^35^ reported a decrease in various raffinose family oligosaccharides (RFO) two days after heat stress priming. In line with this observation, we detected a downregulation of RFO intermediates such as sorbitol/mannitol, raffinose across different days of the HSM phase (Fig. 3c,d). Overall, our multi-omics dataset is in line with the previous findings and offers a valuable resource for future in-depth analyses of plant responses to heat stress priming and memory.

### Statistical analysis

Statistical analyses were performed using GraphPad Prism (GraphPad Software Inc., San Diego, CA). Experimental data were analyzed with R Studio and Excel.

## Supplementary information

Preliminary outputs from different omics datasets

## Data Availability

In this study, we generated for the first time a large multiomic dataset of the Arabidopsis heat stress memory phenotype across different time points after the heat stress priming stimuli and their control conditions. Using high-resolution mass spectrometry, measured proteomics and metabolomics data. These datasets and their associated raw files can be accessed through MassIVE repository under the accession MSV000098061, MSV000098074. We employed transcriptomics for these studies, and the files have been deposited (NCBI) Sequence Read Archive (SRA) under accession number SRP589115. The corresponding primary outputs of this analysis have been provided in the attached Excel files.

## Code availability

The commercially available Proteome Discoverer tool was used to analyse the proteomics data (https://www.thermofisher.com/order/catalog/product/PROTEOMEDISC3). For the RNA-Seq the DE-Seq2 (https://bioconductor.org/packages/release/bioc/html/DESeq2.html) was used to analyse the data. The GC-MS files were analysed using the TARGET SEARCH (https://www.bioconductor.org/packages//2.10/bioc/html/TargetSearch.html).
